# Successful treatment of generalized pustular psoriasis with chronic hepatitis B using spesolimab: A case report

**DOI:** 10.1097/MD.0000000000041979

**Published:** 2025-04-25

**Authors:** Ruiyuan Ming, Lei Zhang, Xincai Xiong

**Affiliations:** a Department of Dermatology, Affiliated Hospital of North Sichuan Medical College, Nanchong, China.

**Keywords:** biologics, chronic hepatitis B, generalized pustular psoriasis, spesolimab

## Abstract

**Rationale:**

Generalized pustular psoriasis (GPP) is a severe form of pustular psoriasis, which can endanger the patient life. However, the conventional treatment for GPP presents several limitations.

**Patient Concerns:**

In this report, we present a 54-year-old male patient with GPP and chronic hepatitis B. The use of glucocorticoids and acitretin for GPP caused liver injury in our case.

**Diagnoses:**

The patient was diagnosed with GPP with chronic hepatitis B.

**Interventions:**

The patient accepted traditional therapy, including glucocorticoids and acitretin. The use of glucocorticoids and acitretin for GPP caused liver injury in our case. Therefore, acitretin was discontinued (but not the glucocorticoid). After excluding contraindications, the patient was administered a single dose of spesolimab (900 mg).

**Outcomes:**

The rash resolved within 48 hours after the patient was administered a single dose of spesolimab (900 mg).

**Lessons:**

Our study shows that spesolimab could be employed as a safe and effective therapeutic option for patients with GPP and other underlying conditions.

## 1. Introduction

Generalized pustular psoriasis (GPP) is a rare, potentially life-threatening dermatosis triggered by stress, glucocorticoid withdrawal, pregnancy, or infection. GPP is characterized by an acute eruption of macroscopically visible primary sterile pustules on an erythematous base, with a potentially life-threatening systemic inflammation. It is not limited to only acral skin or psoriasis plaques. Spesolimab, an anti-IL-36 receptor monoclonal antibody, has been reported to achieve both rapid and sustained control of GPP flare symptoms. However, no published case is yet available on using spesolimab in patients with GPP and chronic hepatitis B.

## 2. Case report

**Figure 1.**
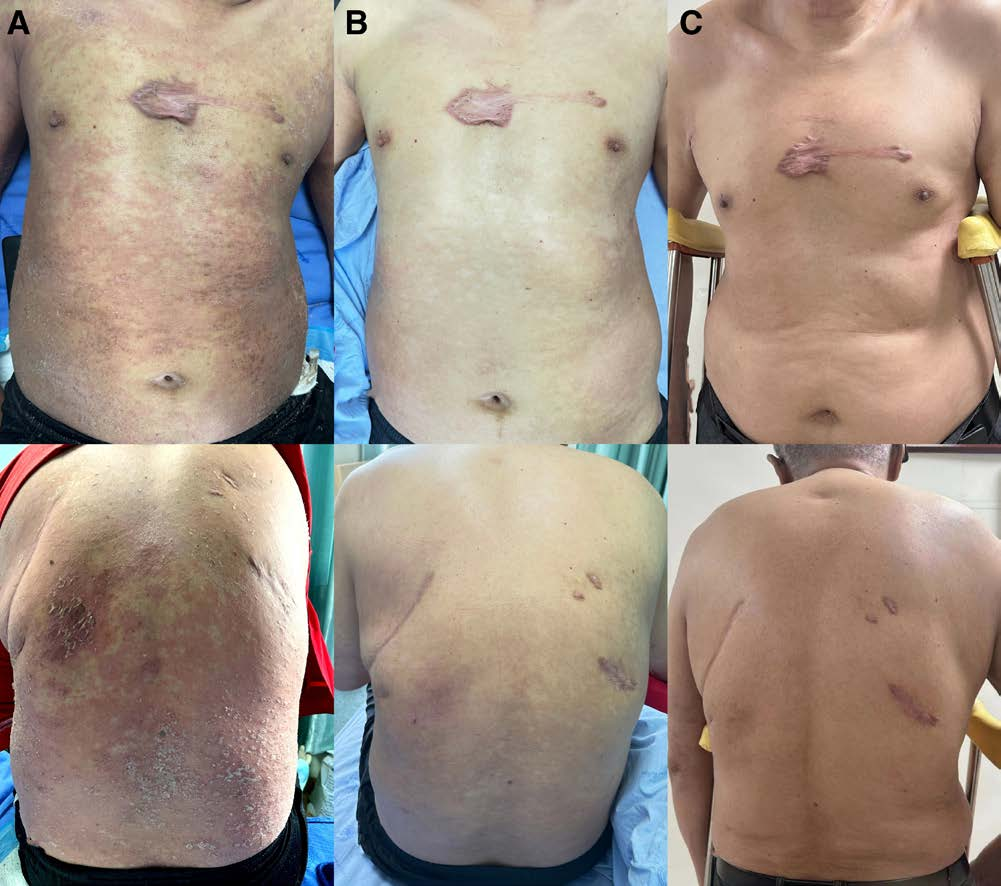
(A) Generalized edematous erythema with numerous pustules over the body. (B) The lesions were significantly improved 48 hours after the application of spesolimab. (C) The lesions almost disappeared 1 month after the application of spesolimab.

A 54-year-old man presented to our hospital with a complaint of recurrent erythema, papules, pustules, and scales with pruritus for 15 years, with a recurrence for 1 week. The patient had experienced scattered erythema, plaques, White scales, and pruritus on his extremities for 15 years. He has a history of hepatitis A and B, hypertension, type 2 diabetes, and chronic obstructive pulmonary disease. The patient underwent surgery for pleurisy and internal fixation of a left tibial fracture. One week before he came to us with his complaint of recurrent erythema, he was admitted to our hospital for the treatment of a left tibial fracture. During the course of the treatment, the patient developed fever and chills, with the highest temperature reaching 39.5°C. Diffuse erythematous rashes appeared on his body, with numerous pustules and scales (Fig. 1A). No obvious abnormalities were noted on the system examination.

**Figure 2.**
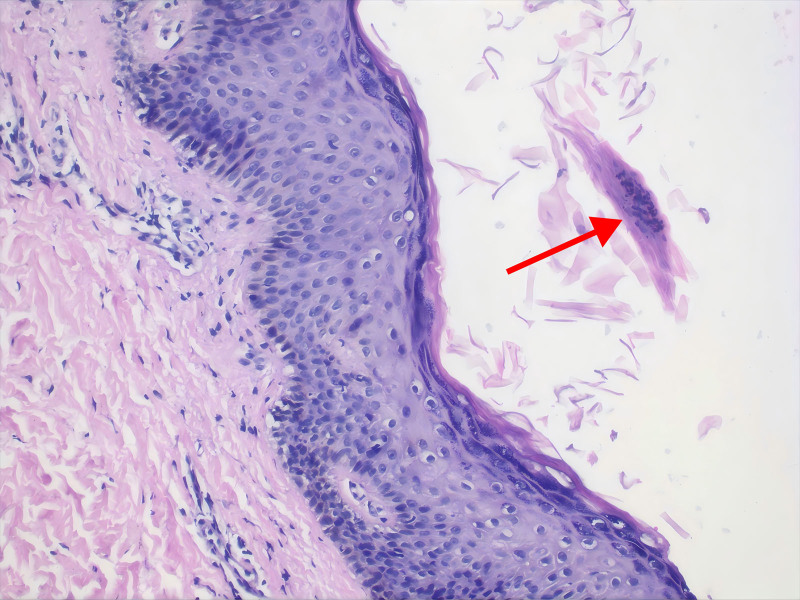
Histopathological examination of a punch biopsy specimen. Epidermal net-like keratinization with irregular keratinization, small clusters of Munro pustules, thickened stratum corneum, and a few pigmented cells in the basal layer were observed. The arrow points to Munro microabscess (hematoxylin & eosin; original magnification ×100).

The diagnosis of GPP was confirmed. His GPP Physician Global Assessment10 (GPPGA) was 4, while his GPP Area and Severity Index11 (GPPASI) was 67.2. A skin biopsy conducted on the patient revealed epidermal net-like keratinization with irregular keratinization, small clusters of Munro pustules, a thickened stratum corneum, and a few pigmented cells in the basal layer (Fig. 2).

The patient was initially treated with both glucocorticoids and acitretin for psoriasis. However, the rash did not subside. In addition, liver function tests showed liver damage. Therefore, acitretin was discontinued (but not the glucocorticoids). After excluding contraindications, the rash resolved within 48 hours after the patient was administered a single dose of spesolimab (900 mg) intravenously (Fig. 1B). The patient also exhibited improvement in pustules within 48 hours of administering spesolimab. The GPPASI score became 0.2, and the GPPGA score became 0. The histopathological examination suggested that the administration of spesolimab significantly reduced the accumulation of neutrophils in the lesion than that observed before its administration. Laboratory examination revealed no exacerbation of hepatic injury. In addition, no increase in the activity of the hepatitis B virus and no alleviation of inflammation were observed. Before spesolimab administration, the amount of aspartate aminotransferase was 168 U/L (normal range 15–40 U/L), which decreased to 58 U/L 5 days after using spesolimab and drugs (glycyrrhizin and glutathione) for protecting the liver. The concentration of alanine aminotransferase was 295 U/L (normal range 9–50 U/L), which decreased to 96 U/L after administering spesolimab and drugs for protecting the liver for 5 days. The quantitative detection of HBV–DNA was 2.56^E+01^ IU/mL (normal range, <2.0^E+01^ IU/mL) before treatment and <2.0^E+01^ IU/mL after treatment with spesolimab and anti-hepatitis B virus drugs for 48 hours. The C-reactive protein reduced from 74.95 to 23.54 mg/L in 10 days. After treatment with spesolimab for 1 month, the patient lesions had reduced to only a few scales without pustules (Fig. 1C). Importantly, he did not experience any side effects or recurrences during the treatment.

## 3. Discussion

GPP is a severe form of psoriasis that can become life-threatening. The exact cause and pathogenesis of pustular psoriasis have not yet been fully elucidated, although it may involve a combination of genetic and immune dysregulation factors. GPP is associated with the IL-36 signaling axis. The IL-36 subfamily comprises 3 pro-inflammatory agonists (IL-36α, IL-36β, and IL-36γ), their receptor (IL-36R), and one antagonist (IL-36Ra). The proinflammatory ligands IL-36 (α, β, and γ) exert intracellular responses through IL-36R, which is expressed on keratinocytes, dendritic cells, macrophages, neutrophils, and T helper cells.^[1]^ The activation and upregulation of IL-36R result in a downstream signaling cascade mainly driven by IL-1, IL-6, IL-8, and TNF-α/IL-17A.^[2]^ This cytokine storm attracts a neutrophilic infiltrate to the epidermis and leads to neutrophil-driven inflammatory responses.^[3]^

Spesolimab is an anti-IL-36 receptor monoclonal antibody. It can quickly and sustainably control the symptoms of GPP.^[4]^ Spesolimab inhibits IL-36R signal transduction by specifically binding to IL-36R, which decreases the downstream inflammatory cascade as well as the recruitment of neutrophils and other immune cells.^[2]^ As a result, it can efficiently treat GPP.^[5]^ A recent meta-analysis on 329 patients from 16 studies evaluated the comparative efficacy of multiple biologic agents in the treatment of GPP, and demonstrated that, compared to IL-17 inhibitors, IL-23 inhibitors, and TNF-α inhibitors, IL-36 inhibitors deliver a faster and more substantial clinical response.^[6]^ In the past 2 years, a series of cases, including some special ones, have proven the efficacy and safety of spesolimab. For example, Müller and Kreuter reported a case of hypertension and ulcerative colitis, in which the use of spesolimab did not worsen the condition of the patient.^[7]^ Yang et al reported the case of a pregnant patient with GPP, who gave birth to a healthy baby after using spesolimab.^[8]^ Wen et al reported improvement in the condition of a patient with GPP and Acrodermatitis continua of Hallopeau after receiving spesolimab.^[9]^ Matsuo et al reported a patient with GPP exhibiting a discrepant resolution time between the appearances of rashes.^[10]^ Minami et al reported a patient with systemic lupus erythematos (SLE) whose condition did not exacerbate after the administration of spesolimab.^[11]^ In conclusion, while spesolimab has shown potential, its broader clinical application necessitates further investigation because of the current paucity of large-scale clinical data. Future research should focus on conducting larger-scale randomized controlled trials and long-term follow-up studies to comprehensively assess its efficacy and safety. More importantly, no case of spesolimab use in GPP patients who also have chronic hepatitis B has yet been reported. There is no clinical evidence to prove that hepatitis B virus will not become active after using spesolimab.

We herein reported a patient with GPP who also had a history of hepatitis A and B, hypertension, type 2 diabetes, and chronic obstructive pulmonary disease. During the course of the illness, the patient experienced recurrent fever symptoms. In addition, the patient developed acute liver injury while using acitretin and glucocorticoid for GPP.

Spesolimab is a humanized IgG1 monoclonal antibody that is expected to metabolize in the body similarly to endogenous IgG. It degrades into small peptides and amino acids through the degradation metabolic pathway. As spesolimab does not undergo metabolism by the liver, it will not exacerbate liver injury. The contraindications for spesolimab are hypersensitivity and active infections, such as active tuberculosis.

This is the first case of successfully using spesolimab to treat a refractory GPP patient with hepatitis B infection and compromised liver function who could not tolerate other treatment options. After the patient received Spesolimab treatment, the skin lesions showed rapid and remarkable improvement. In addition, the hepatic function remained stable with no evidence of reactivation or active replication of the hepatitis B virus. No adverse events were reported. This case supplements the real-world data on the use of spesolimab outside of the study exclusion criteria. It provides a safe and effective treatment option for GPP patients with active HBV and poor liver function. Hence, spesolimab may present a safe and efficacious alternative therapeutic option for patients with GPP who have other underlying conditions as well.

## Acknowledgments

We thank the patient for participating in the study.

## Author contributions

**Conceptualization:** Xincai Xiong.

**Data curation:** Ruiyuan Ming, Lei Zhang.

**Investigation:** Ruiyuan Ming.

**Project administration:** Xincai Xiong.

**Supervision:** Xincai Xiong.

**Writing – original draft:** Ruiyuan Ming, Lei Zhang.

**Writing – review & editing:** Lei Zhang, Xincai Xiong.
